# Calcium and vitamin D supplementation effects on metabolic factors, menstrual cycles and follicular responses in women with polycystic ocvary syndrome: A systematic review and meta-analysis

**DOI:** 10.22088/cjim.10.4.359

**Published:** 2019

**Authors:** Zahra Shojaeian, Ramin Sadeghi, Robab Latifnejad Roudsari

**Affiliations:** 1 Department of Midwifery, School of Nursing and Midwifery, Mashhad University of Medical Sciences, Mashhad, Iran; 2 Nuclear Medicine Research Center, School of Medicine, Mashhad University of Medical Sciences, Mashhad, Iran; 3 Nursing and Midwifery Care Research Centre, Mashhad University of Medical Sciences, Mashhad, Iran

**Keywords:** Calcium and vitamin D, Polycystic ovary syndrome, Metabolic factors, Manstrual cycle

## Abstract

**Background:**

Polycystic ovary syndrome (PCOS) is the most popular endocrine disorder in reproductive age with unknown etiology and many comorbidities. This systematic review focused on the effectiveness of calcium and vitamin D (Ca/ Vit.D) supplementation on metabolic factors, menstrual cycles, and follicular responses in PCOS patients.

**Methods:**

Relevant studies were identified from the following electronic databases including Pub Med, Web of Science, Cochrane Central Register of Controlled Trials (CENTRAL), The Cochrane Library, issue Oct. 2018, Evidence Based Medicine Reviews (EBMR), Google Scholar, EMBASE, as well as Farsi databases including Magiran and SID from 2000 to 2018. Out of 449 articles, six clinical trials met the inclusion criteria.

**Results:**

Out of six studies included in the study, only three studies had sufficient data for meta-analysis. Overall, studies showed that prescribing Ca/ Vit. D supplementation with metformin improved menstrual regularity and follicular maturation and significant decreases in serum insulin levels, homeostasis model of assessment-insulin resistance (HOMA-IR) and fasting blood sugar (FBS) and also significant rises on quantitative insulin sensitivity check index (QUICKI) at two studies. Hirsutism and level of testosterone decreased significantly after adding Ca/ Vit. D to metformin in two different studies. Based on two different trials, co-supplementation of Ca/ Vit. D had a significant impact to decrease serum triglycerides and VLDL-cholesterol levels as well as levels of cholesterol and LDL.

**Conclusion:**

It is possible that calcium and vitamin D supplementation improve menstrual disturbances and metabolic factors in PCOS in a long-term period, but further trials are still needed to confirm these findings.

Polycystic ovary syndrome (PCOS) is one of the most heterogeneous endocrine disorders in reproductive age affecting approximately 5-10% of females. PCOS is a chemical or clinical hyperandrogenism associated with chronic anovulation and polycystic ovary. The clinical features of PCOS include infertility, hyperandrogenism, obesity, abnormal glucose metabolism, insulin resistance (IR), hirsutism and acne vulgarism (1). PCOS prevalence in Iran was reported according to NIH and Rotterdam criteria 6/8% and 19/5%, respectively, in 2014 (2). The causes of PCOS are unknown. 

It is hypothesized that insulin resistance leads to hyperinsulinemia and as a result , increased insulin promotes secretion of androgens from the ovaries, which itself decreases the amount of serum sex hormone binding globulin (SHBG), therefore, serum free testosterone is elevated and ovarian follicle growth and maturation is altered by hyperinsulinemia and androgen excess (3). Women with PCOS may be at elevated risk of vitamin D deficiency (VDD). VDD has been observed in approximately 67-85 percent of women with PCOS (4). Positive associations of VDD with some well- known co morbidities of PCOS including typ2 diabetes, insulin resistance, metabolic syndrome and cardiovascular disease has been reported (5, 6). 

Direct effect of calcium and vitamin D on the ovarian and/or adrenal steroid genesis pathway may be justified by the observed reduction in circulating androgens (7). Some studies reported positive correlations between total serum calcium with insulin level and insulin resistance and fasting serum glucose in the larger healthy population. 1,25 (OH) 2D likely has effect on insulin secretion by the significant rise in intracellular ionic calcium level following 1,25(OH)2D-stimulated secretion of insulin by islet cells (8). A significant decrease level of vitamin D; has been reported also, glucose and phosphorus level in overweight obese women with PCOS correlated negatively with insulin and insulin resistance and positively with vitamin D level (8).

In accordance with limited evidence, calcium and vitamin D metabolism affects oocyte maturation and production of androgens (9). In Razavi’s study (2016), prescribing vitamin D-K-calcium co-supplementation for 8 weeks among vitamin D-deficient women with PCOS has beneficial effects on serum dehydroepiandrosterone sulfate (DHEAS), free testosterone, plasma total antioxidant capacity (TAC), and malondialdehyde (MDA) concentrations (MDA) levels (10). 

Ott et al. (2012) found that parathyroid hormone (PTH) reversely correlated with serum calcium and 25OHD3, whereas positive correlations were found between PTH and body mass index as well as testosterone (11). Foroozanfard (2015) showed beneficial effects of calcium plus vitamin D co-supplementation for 8 weeks among overweight and vitamin D-deficient women with PCOS on inflammatory factors and biomarkers of oxidative stress (12). Some evidence has shown that vitamin D and calcium metabolism affects oocyte maturation and production of androgens but other studies have not agreed with these findings. There is a dearth of systematic review and meta-analysis on the effect of Ca/Vit D on menstrual cycles and metabolic factors in PCOS patients. No systematic review has also been conducted in this field in Iran. The main mechanism of Ca/ Vit. D effects have not yet been determined. Considering the lack of research and the validity of the results, this study reviews the current evidence and makes recommendations about the effect of calcium and vitamin D supplementation on metabolic factors, menstrual cycles and follicular responses in PCOS patients.

## Methods

**Fig1:**
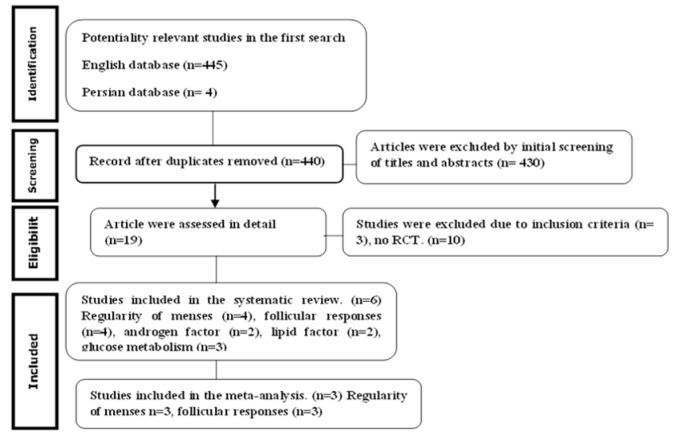
Search strategy for systematic review

**Literature Search strategy: **Relevant studies were identified from the following electronic databases: Pub Med, Web of Science, Cochrane Central Register of Controlled Trials (CENTRAL), The Cochrane Library (issue 7, 2016), Evidence-Based Medicine Reviews (EBMR), Google scholar, EMBASE, Magiran and SID using keywords of polycystic ovarian syndrome, vitamin D, calcium compounds, calcitriol, and the combination of these keywords from 2000 to October 2018. Cited references of retrieved trials as well as systematic reviews were manually screened to identify any further relevant trials which was possibly missed in electronic search. Also, the authors were contacted for additional missing data. Out of 449 relevant published trials, six RCTs met the inclusion criteria and were reviewed systematically (figure 1) (13). 

**Inclusion and exclusion Criteria: **Studies were considered to be included in systematic review if they met all of the following criteria:

Designed as randomized controlled trials (RCTs). 

Evaluated the impact of calcium plus vitamin D supplementation in PCOS patients.

Measured outcomes include menstrual cycle regularity, follicular response and metabolic factors. 

Studies were excluded if they were short communications and case reports/ series as well as publications from meetings/conferences or studies which did not provide baseline descriptions of metabolic parameters.

**Data extraction: **Two reviewers independently assessed and extracted the data using an arranged checklist. The extracted data included the first author, sample size, the age of subjects, diagnostic criteria for PCOS, criteria for exclusion, study design, intervention, the time period of intervention and the outcome measures (metabolic factors, regularity of menses and follicular responses) (table 1). Disagreements between reviewers were resolved by consensus and arbitration.

**Table1:** Characteristics of 6 clinical trials included in systematic review

**Key findings**	**Intervention period and type of intervention**	**Inclusion and exclusion criteria**	**Sample size**	**Age**	**Year of data collection**	**Author/** **Reference/** **Year of publication**
Regularity of Menes and pregnancy rate had no significant difference in three groups after treatment and Follicular response was not significant between prescribing Ca/ Vit. D or metformin alone, though relatively was higher in Group 1 compared with Group 3(p = 0.29). Frequency of response to treatment in Group 2 was higher than in the other two groups during the fifth and sixth months of the follow-up period.	Intervention period: 3 months Divided into three equal groups Group 1 received 1,000 mg of calcium and 400 IU of vitamin D per day Orally. Group 2 received the same as Group 1, plus 1,500 mg/day of metformin. Group 3 received 1,500 mg/day of metformin.	Inclusion: Rotterdam criteria^1^ Exclusion: systemic diseases such as Cushing’s syndrome, hyperparathyroidism or hyperprolactinemia, androgen secreting tumors, history of abdominal/pelvic surgery, coexisting male factor infertility, or abnormal hysterosalpingography	N=60 sample in three group	20-40	2004 Tehran	1- Batool Rashid et al(13) 2009
Prescribing Ca/ Vit. D in group3, compared with other groups, led to decreased serum insulin levels (P < 0.03), HOMA-IR score (P <0.04) and a significant rise in QUICKI index) P < 0.001)**,** significant decrease in serum triglycerides) P < 0.02) and VLDL-cholesterol levels (P <0.02), but had no significant effects on FPG, total LDL, HDL, and non-HDL-cholesterol levels. Adjustment for age and baseline BMI did not affect findings except for HOMA-IR score (P<0.05).	Intervention period : 8 weeks four groups to receive: 1) CALCIUM:1000 mg/d calcium ‏ vitamin D placebo (n = 26); 2) VITD:50,000 IU/wk vitamin D ‏ calcium placebo (n = 26) 3) CA+vit D:1000 mg calcium/d ‏ 50,000 IU/wk vitamin D (n = 26) 4) placebo:calcium placebo ‏ vitamin D placebo (n = 26)	Inclusion: Rotterdam criteria Exclusion: aged<18 or >40 years, those with BMI < 25 kg/m2, individuals with neoplastic, hepatic, renal or cardiovascular disorders, malabsorptive disorders, those taking calcium and vitamin D within the last 6 months and women who had calcium intake more than 1500 mg per day using hormone therapy, antidiabetic, or anti-obesity medications within the last 6 months intended to adopt a diet and/or a specific physical activity program	N=104 overweight and obese vitamin D deficient women	18-40	2013 kashan	2- Zatollah Asemi(18) 2014
No Significant difference between groups before and after treatment in Cardio metabolic risk factor(FBS, LDL, cholesterol, systolic and diastolic blood pressure, BMI,TG. and just significant increased HDL in Ca/ Vit. D and metformin group and testosterone in metformin group. There was significant deference between groups after treatment in many factors such as BMI, BP, FBS, LDL, and Cholesterol (p<0.001). There was no statistically difference between rates of changes in the parameters under study in the patients with and without metabolic-syndrome.	Intervention period: 12 weeks The selected individuals were divided in simple random form into 2 groups First group: under treatment with 1000 milligrams of Calcium and Vit-D-400 IU twice a day. Plus metformin1500 mg/day Second: : metformin1500 mg/day	Inclusion: Rotterdam criteria Exclusion: History of any underlying and chronic disease (chronic kidney disease, etc.), history of abdominal and pelvic surgery, abnormal hysterosalpingography, abnormal serum prolactin level, smoking, pregnancy, current or previous Statin use during the past 2 months, insulin use, use of corticosteroids, anti-obesity medication, and history of neoplastic diseases.	N=72	20-45	2015 Hamadan	3Mohsen Gharakhani(17), -2015
There was no statistically significant difference in folliclar size between groups (p=0.1) and the first and second month (p=0/82). Average size follicles in the first, second and third months was higher in intervention group compared with The control group (p<0/05) and size of follicles in the third compared with the second months was significantly different (p=0/01).Therefore adding Ca/ Vit. D to the usual treatment with clomiphene increased the size of follicles, especially after the second menstrual cycle.	Intervention period : 3 months Patients randomized blocking the two groups (n = 22) and The control group (22 cases).The intervention group received clomiphene citrate 50 mg tablets with 400 IU of vitamin D3 And 1,000 mg of calcium and 50 mg clomiphene daily for the control group was given Plus. Placebo. For these patients, ultrasound up to three cycles, and every once in a 13-day menstrual cycle was conducted to evaluate the size of the dominant follicle and if Not positive pregnancy test, clomiphene dose in the second month Double treatment (100 mg) and in the third month treatment Three times (150 mg) increased	Inclusion: Rotterdam criteria Exclusion: Endocrine disease for example Cushing's and hypothyroidism, hyperthyroidism, increased blood prolactin	n=44 infertile women.	20-43	2011 kermanshah	4- Robabeh Mohammad beige(15) 2012
The presence of Ca/ Vit. D caused frequency of regular menstrual cycles and dominant follicle were significantly higher in groups 1 and 2 than others (P < 0.05) but frequency of acne, hirsutism and BMI were not significant among different groups.	Intervention period: 4 months 1. Metformin group (group1); received 1500 mg/day metformin 500 mg) (n = 20). 2. Metformin plus calcium and Vitamin D (group 2); received 1500 mg/day metformin plus 1000 mg/day calcium 500 mg) plus 50,000 IU/2 weeks pearl Vitamin D3 (pearl vitamin D3 50000 IU) (n = 20. ( . 3. Calcium and Vitamin D (group 3); received 1000 mg/ day calcium plus 50,000 IU/2 weeks pearl Vitamin D3 (n = 20). Placebo group (group 4) received two low calorie sweetener tablets per day (n = 20).	Inclusion: NIH 1990 criteria^2^ Exclusion: Cushing syndrome, renal failure (Cr ≥1.5), androgenic ovarian and adrenal tumor, primary and secondary hyper parathyroidism, osteomalasia, cancers, gasterectomy, mental retardation, anticonvulsive drugs use and congenital adrenal hyperplasia	N=80	20-40	2013 Isfahan	5- Hatav Ghasemi Tehrani(16), 2014
The mean levels of HOMA in group 1 and mean levels of QUICKI in group 2 were significantly higher than in other groups. The percentage and the mean diameters of the dominant follicles were higher in group 2 than in group 1. Also, group 2 showed lower rate of irregular menstruation after treatment than group 1 but without significant difference. Therefore calcium and vitamin D plus metformin are useful in treatment of PCOS via modifying insulin and causing decreased insulin resistance and increased insulin sensitivity.	Intervention period: 3months Women in group 1 (Metformin only group) received Glucophage XR 1000 mg once daily, orally and diet. -Women in group 2 (Metformin, vitamin D and calcium group) received Glucophage XR1000 mg once daily ,orally as above, plus extreme 500 mg calcium carbonate + 200 Iu vitamin D3 twice daily and diet.	Gynecological and ultrasound examination with general criteria of PCOS (hirsutism, obesity, etc.).	N=120 women with PCOS	30/2-30/5	2015 Egypt	6-Mohamed A.Albahi (19) 2015

1-According to the Rotterdam criteria the presence of two of the three following characteristics was required for inclusion in the study for PCOS: (1) oligomenorrhea/amenorrhea, (2) chemical or clinical findings of hyperandrogenism, and (3) polycystic ovaries on transvaginal Sonography

2- NIH 1990 criteria1-(Chronic anovulation, 2. Clinical or biochemical evidence of hyper androgenism and 3. Roll out of other causes (thyroid stimulating hormone, follicle-stimulating hormone, and prolactin and testosterone measurement).

**Figure 2:**
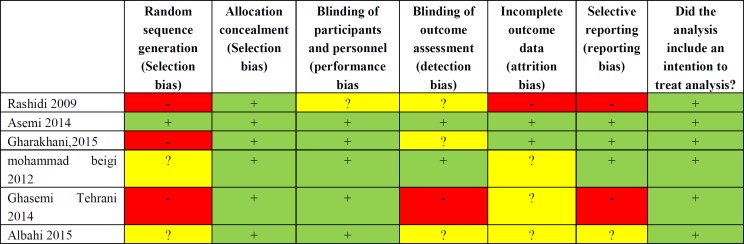
Risk of bias summary: Systematic review. Authors’ judgments of each risk of bias item for each included study

**Figure 3:**
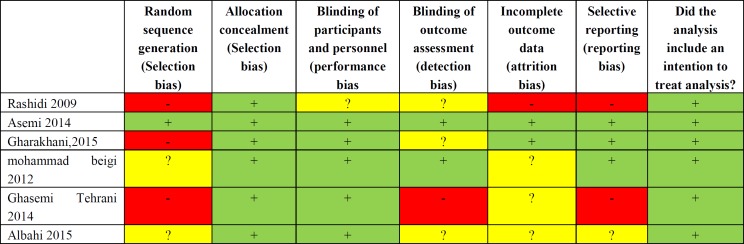
Risk of bias graph systematic review. Authors’ judgment of each risk of bias presented as percentage across all included studies

***Quality assessment: ***The quality of the included studies were evaluated by Oxford Center for Evidence-Based Medicine checklist for RCTs. This tool consists of six general questions including the way of patient's assignment, similarity, and matching of groups, equality of allocated treatment, loss to follow-up and intention-to-treat analysis, blindness and effect size which was assessed with three answers of yes, no and unclear. Risks of bias assessment are shown in figure 2. Two authors independently assessed the risk of bias for each study using the criteria outlined in the Cochrane Handbook for Systematic Reviews of Interventions (fig 3) (14).

**Data synthesis: **We interpreted the results using two effect sizes, odds ratio (OR) and risk difference (RD). For heterogeneity evaluation, Cochrane Q test (p<0.05 as statistically significant) and I^2^ index were used. According to studies difference, random effects models of analysis were used (heterogeneity was not significant, p>0.05; I2=0%). The latter was used to assess how much of the variance across studies is likely to be real and is not due to sampling errors. 

All statistical analyses were done by Comprehensive Meta-analysis Version 2 (Biostat, Englewood, NJ, USA). Subgroup analysis was performed on metabolic factors.

## Results

**Figure 4:**
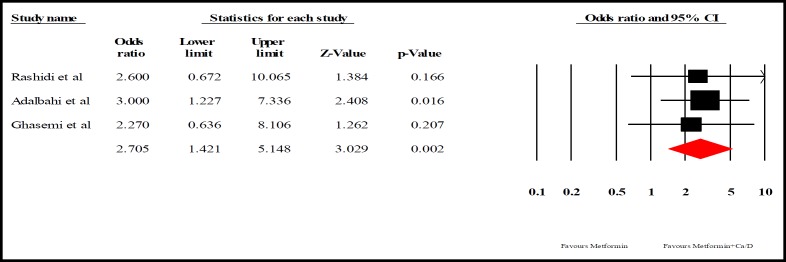
Effect of adding Ca/ Vit. D to metformin on regularity of menses based on Odds ratio. The horizontal lines denote the 95% CI

Out of 449 relevant published trials, six RCTs met the inclusion criteria. All of the six articles were randomized controlled trials designed to evaluate the effect of Ca/ Vit. D co-supplementation for PCO patients. This study was conducted on 435 PCOS women. Five studies defined PCOS based on criteria of Rotterdam and one study diagnosed PCOS using the National Institutes of Health criterion. Four studies were conducted in Iran and one study in Egypt. Four of six studies compared women who received metformin with or without Ca/ Vit. D. This study included one study compared regularity of menses and number of large follicles (15); another compared regularity of menses, dominant follicle and hyperandrogenism (16) and one study compared metabolic factors and another regularity of menses, follicle dominant and glucose metabolism parameters. Two other studies, one study compared metabolic parameters between different groups receiving ca or vitamin D or both or none of them. One study compared distance between menses and follicle growth in women receiving clomiphene with or without Ca/ Vit. D (15). The summarized characteristics of the included studies are shown in table 1. Three of four RCTs, reported regularity of menses and follicular responses. One of four RCTs did not include in meta-analysis due to the differences in study design. Figure 3 shows the forest plots of the meta-analysis on the regularity of menses in studies with co-supplementation of Ca/ Vit. D with metformin. As shown in figure 3, the effect of adding Ca/ Vit. D to metformin on regularity of menses was higher than ca/D only (OR2.075 95%; CI 1.421 to5.148], P =002; heterogeneity I2=0.000 %; P =0.938). Beigi (2012) used Mann Whitney test to assess the differences between the group receiving Ca/ Vit. D versus placebo in addition to clomiphene (15). No statistically significant difference was observed between the average duration of menstruation (p=0.7) and the distance between periods (p=0.5) in two groups. Figure 4 shows the forest plots of the meta-analysis on follicular responses in three studies. 

**Figure 5:**
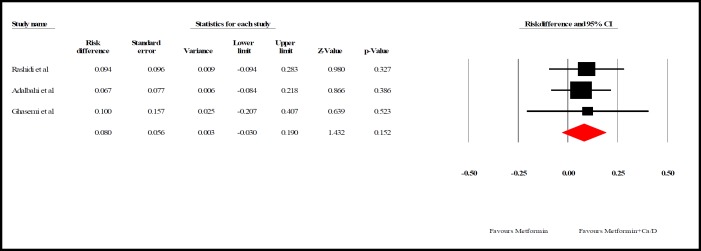
Effect of adding Ca/ Vit. D to metformin on follicle response based on risk differences. The horizontal lines denote the 95% CI

As shown in figure 5, the effect of adding Ca/ Vit. D to metformin on follicle response was higher than Ca/ Vit. D only but the difference was not significant ((RD, 0.080, 95%; CI -0.030 to0.190], P=0.152; heterogeneity I2=0.000 %; P=0.152). Beigi (2012) compared two groups with Mann Whitney test after 3-month-use of clomiphene citrate (cc) combined with calcium and vitamin D or placebo. Changes of follicular size was not significant (P=0.1) but the mean of follicular size in the first, second and third month following intervention was higher in the group who received Ca/ Vit. D compared to placebo group (P=0.03, P=0.01, P=0.003) (15).

Metabolic factors were not included in meta-analysis due to differences in study design (17, 18). Co-supplementation during 8 weeks with calcium and vitamin D or adding Ca/ Vit. D with metformin for 12 week resulted in a significant reduction in serum insulin levels (p=0.03, p=0.015) and HOMA-IR score (p=0.04, p=0.001) and a significant elevation in QUICKI index (p=0.001). Administration of calcium and vitamin D alone had no significant effect on total FPG (fasting plasma glucose) (p=0.08) (18) but along with metformin, it had significant reduction (p=0.002, p<0.001)) (19, 17). The level of androgenic factors was measured by measuring hirsutism or the level of testosterone and DHEAS. Hirsutism and the level of testosterone decreased significantly after adding Ca/ Vit. D to metformin in two different studies (P>0.05, p=0.037) (15, 16); but changes in the level of DHEAS was not significant (p=0.987) (17). Calcium-vitamin D co-supplementation in PCOS women resulted to a significant decrease in serum triglycerides (P=0.02) and VLDL-cholesterol levels (P=0.02) compared with other groups who had calcium or vitamin D alone. However, it had no significant effect on LDL, HDL, and non-HDL-cholesterol levels. (18). Gharakhani (2015) found that adding Ca/ Vit. D with metformin resulted in significant decrease in the levels of cholesterol and LDL (P<0.001) (17).

## Discussion

The present review supports that serum calcium and vitamin D status affect the metabolic and menstrual cycle changes and follicle maturation in PCOS patients the important sources of heterogeneity, according to meta-regression findings: the average duration of menstruation and the distance between periods were not important sources of heterogeneity. The other potential sources of heterogeneity in PCOS, regularity of menses and follicular responses were assessed. 

Metabolic factors were not included in meta-analysis due to differences in study design. Subgroup metabolic factor analysis showed that the improvement of hirsutism and the levels of testosterone, cholesterol and LDL after adding Ca/ vit D. It seems that the effectiveness of clomiphene citrate or metformin in combination with calcium and vitamin D in improving menstrual regularity and follicular maturation is higher than using only metformin or clomiphene. In an observational study, prescription of vitamin D with calcium therapy resulted in normal menstrual cycles within 2 months in nine of thirteen women, from which two women became pregnant (20). Firouzabadi (2012) reported that 100 000 IU vitamin D for 6 months and 1 000 mg/day calcium co-administration among PCOS cases resulted in improved follicular maturation, regularity of menses, and androgen related symptoms especially in women with vitamin D deficiency (21). Across studies within Alisa review (2013), several studies suggested that calcium metabolism dysfunction is associated with impaired oocyte development. 

As a result, vitamin D deficiency may alter the availability and function of calcium, suggesting that co-supplementation of vitamin D could correct this imbalance. Menstrual irregularity is improved by co-supplementation of Ca/ vit. D (22, 23).

Using Ca/ vit. D with metformin decreased serum testosterone and hirsutism. In this systematic review, assessing metabolic changes for androgens showed Ca/ vit. D in combination with metformin, have positive effect on androgenic factors and symptoms. Serum DHEAS, testosterone, SHBG and free androgen index (FAI) are used as diagnostic markers of hyperandrogenism. In several studies, significant reduction in androgenic factor was observed compared to baseline values after co- supplementation of calcium and vitamin D (21, 23, 24). Razavi et al. demonstrated that vitamin D-K-calcium co-supplementation among vitamin D-deficient women with PCOS for 8 weeks resulted in a significant reduction in serum-free testosterone and DHEAS, compared to placebo (10). Earlier studies have also shown that estrogens and androgens directly or indirectly affect vitamin D signaling pathways. Vitamin D influences the balance between androgens and estrogens, which may have a role in pathophysiologic mechanisms of PCOS (8). The prevalence of VDD among women with PCOS, BMI, insulin resistance parameter affect steroidogenesis. In this systematic review, the relationship between VDD and BMI was not seen in all of them but Ca/ vit. D co-supplementation had a beneficial effect on insulin resistance parameter.

Ca/ vit. D co- supplementation had also a beneficial effect on glucose metabolism in this review. In the study of firouzabadi (2012) in Yazd, Iran positive effect was seen on insulin resistance after the six-month treatment with metformin and Ca/ vit. D, especially in women with vitamin D deficiency (21). Bonakdaran (2012) showed that prescribing calcitriol for three months has not significant relationship between vitamin D level and fasting blood sugar, blood glucose two hours after 75 gr glucose and insulin level and HOMA-IR after calcitriol (9). Selimoglu et al. (2010) in contrast, showed improvement in HOMA indices within 3 weeks of a single oral mega dose of 300,000 IU D3 in a pilot study on 11 women with PCOS, (25). In an exhaustive medical literature search, which was conducted by Galusha (2013), multiple studies suggested a weak improvement in menstruation regularity, fertility, BMI, insulin resistance, glucose metabolism and hyperandrogenism following CA/Prescription(23). A meta-analysis conducted by Chunla He (2015) revealed an inverse correlation of serum vitamin D concentrations with HOMA-IR, triglycerides, but positive correlation with HDL-cholesterol ratio and QUICKI (26).

 In another study, no correlation was seen between vitamin D deficiency and its severity with complications of PCOs including obesity and insulin resistance in high school girls who had hypovitaminos D (27). Physiologic functions of active vitamin D affect glucose and insulin metabolism. Factors that affect glucose metabolism included follow up duration, BMI before and after treatment, VDD, duration for treatment and diet which has been reviewed in studies with different methodologies.

This review showed that calcium and vitamin D had beneficial effect on some lipid parameters, such as HDL, TG and VLDL. Chunla (2015) and some authors reported that supplementation of vitamin D does not significantly improve metabolic and endocrine parameters (except triglycerides and fasting insulin) (25). Some studies have shown that magnesium-zinc-calcium-vitamin D co supplementation for 12 weeks in PCOS women was associated with a significant reduction in serum TG, VLDL, total-cholesterol concentrations, total-/HDL-cholesterol ratio, but did not influence other lipid parameters (28). Other studies showed that co-administration of Ca/ vit. D significantly improve lipid profile (29, 30, 31). Al-Hakeim (2009) found that the level of serum Lipids is not correlated with the serum calcium (32). Vitamin D regulates adiposity activity which possibly is related to its potential benefit with respect to insulin sensitivity. The effect of vitamin D on serum triglycerides concentrations is still not known (18, 33). In most of reviewed studies diet, parathyroid hormones and vitamin D levels have not been evaluated.

The important limitations in the current study are few studies focused on calcium and vitamin D supplementation conducted with randomized designs. On the other hand, dosage of calcium and vitamin D supplementation and patients’ baseline serum vitamin D levels varied from study to study, thereby; there is uncertainty that all patients were administered with sufficient amount of calcium and vitamin D supplement. Included studies varied in the definitions of PCOS, which may have affected the assessment of the role of calcium and vitamin D on metabolic parameters in PCOS. There was small sample size, inadequate treatment allocation and insufficient duration of the intervention. 

Deficiency of vitamin D was uncertain except in only one study that may affect the effectiveness of the treatment. Also publication bias could be a possible threat for the validity of the results, therefore the findings should be interpreted with cautiousness (34, 35). Further studies with longer duration of the intervention, and larger sample size are required to confirm our findings.

The main finding of this study which is calcium and vitamin D supplementation may improve metabolic parameters and menstrual disturbances in PCOS cases. However, it cannot be ruled out that calcium and vitamin D supplementation may be a minor pathway towards PCOS, given the small intervention studies and the variability of their results. Thus, long-term, and multiple randomized clinical trials are needed to be conducted to determine whether vitamin D and calcium supplementation affect co -morbidities with PCOS and to demonstrate the benefits of calcium and vitamin D supplementation in treatment of women with PCOS.
